# Life Cycle and Genetic Diversity of *Symplocarpus nipponicus* (Araceae), an Endangered Species in Japan

**DOI:** 10.3390/plants7030073

**Published:** 2018-09-11

**Authors:** Seiji Takeda, Yusuke Onishi, Yoshio Fukui, Takanori Ohsako, Nakao Kubo

**Affiliations:** 1 Graduate School of Life and Environmental Sciences, Kyoto Prefectural University, Hangi-cho 1-5, Shimogamo, Sakyo-ku, Kyoto 606-8522, Japan; usijima5208229064@gmail.com (Y.O.); ohsako@kpu.ac.jp (T.O.); nkubo@kpu.ac.jp (N.K.); 2 Biotechnology Research Department, Kyoto Prefectural Agriculture Forestry and Fisheries Technology Center, Kitaina-yazuma Oji 74, Seika, Soraku-gun, Kyoto 619-0244, Japan; 3 Villages at the Source of a River in Mitsuno, Ayabe, Kyoto 623-1133, Japan; zen-fukui.hime@clock.ocn.ne.jp

**Keywords:** Araceae, genetic diversity, life cycle, microsatellite, *Symplocarpus nipponicus*

## Abstract

*Symplocarpus nipponicus*, a member of the Araceae family, is an endangered plant in several prefectures in Japan. For the conservation of this wild species, we investigated the morphology, life cycle, and genetic diversity of three wild populations. By fixed-point observation over several years, we found that it takes at least four years for the plant to set the inflorescences consisting of spadices and spathes, and another two years for it to set mature seeds. To examine the genetic diversity in the wild population, we developed 11 novel microsatellite markers and investigated the genetic variation in three populations in Kyoto Prefecture: Ayabe, Hanase, and Momoi. The Ayabe population carried less genetic variation than the other two areas, suggesting the isolation of the habitat and thus a higher risk of extinction. Our results provide basic knowledge of the ecological aspects of *S. nipponicus*, as well as molecular techniques for the assessment of its genetic diversity, and thus are useful for the conservation of this endangered species.

## 1. Introduction

*Symplocarpus* is the genus of perennial plants in the family Araceae, including three species found in Eastern Asia [*S. renifolius* Schott ex Tzvelev (syn. *S. foetidus* var. *latissimus* Makino ex H. Hara), *S*. *nipponicus* Makino, and *S*. *nabekuraensis* Otsuka & K. Inoue], one in North America [*S. foetidus* (L.) Salisb. ex W.P.C. Barton], and one in the Far East of Russia [*S. egorovii* N. S. Pavlova & V. A. Nechaev] [1,2,3]. Among the common characteristics of this genus, the leaves emerging from the base of the root have long petioles and inflorescence that consists of a spadix and a dark purple spathe, although the leaf shapes, inflorescence sizes, and pollen shapes are clearly different [1,3]. The flowering season is either spring (*S. foetidus*) or summer (*S. nipponicus* and *S. nabekuraensis*), and their flowers emanate an offensive stench to attract pollinators [1]. The phylogeny of the genus *Symplocarpus* has been examined by chloroplast DNA variation, suggesting that the genus is monophyletic and that *S. nipponicus* is the most ancestral species within the genus [2,4,5]. Interestingly, *S. renifolius* and *S. foetidus* formed a clade in spite of great geographical distances between their populations [2]. *S. nabekuraensis* belongs to the same clade of *S. renifolius* rather than *S. nipponicus* [5].

*S. nipponicus* is an endangered plant species in several prefectures in Japan, and is classified as critically endangered (CR) in Kyoto Prefecture. The habitats are seriously limited, and the procession of inbreeding within a population may be causing the loss of genetic diversity, which is an important factor to recognize how a particular population is endangered [6]. There are several approaches to assess the genetic diversity of a wild population. Microsatellite, which is also called a simple sequence repeat (SSR), is a tandem repeat of DNA sequences existing in the genome of various organisms [7]. The difference in the length of repeat sequences among individuals can be used as a genetic marker, which is detected easily by polymerase chain reaction (PCR) and gel electrophoresis. As a result, microsatellite markers have been widely used for the conservation of various organisms [8]. In *Symplocarpus*, phylogenetic analyses with chloroplast DNA variation have been reported as shown above, although they sometimes cannot distinguish the intraspecific variation [4,5]. Here, we developed the 11 novel microsatellite markers for *S. nipponicus* and assessed the intraspecific genetic diversity of three populations in Kyoto Prefecture.

## 2. Results

### 2.1. Life Cycle and Morphology of S. nipponicus

**Figure 1:**
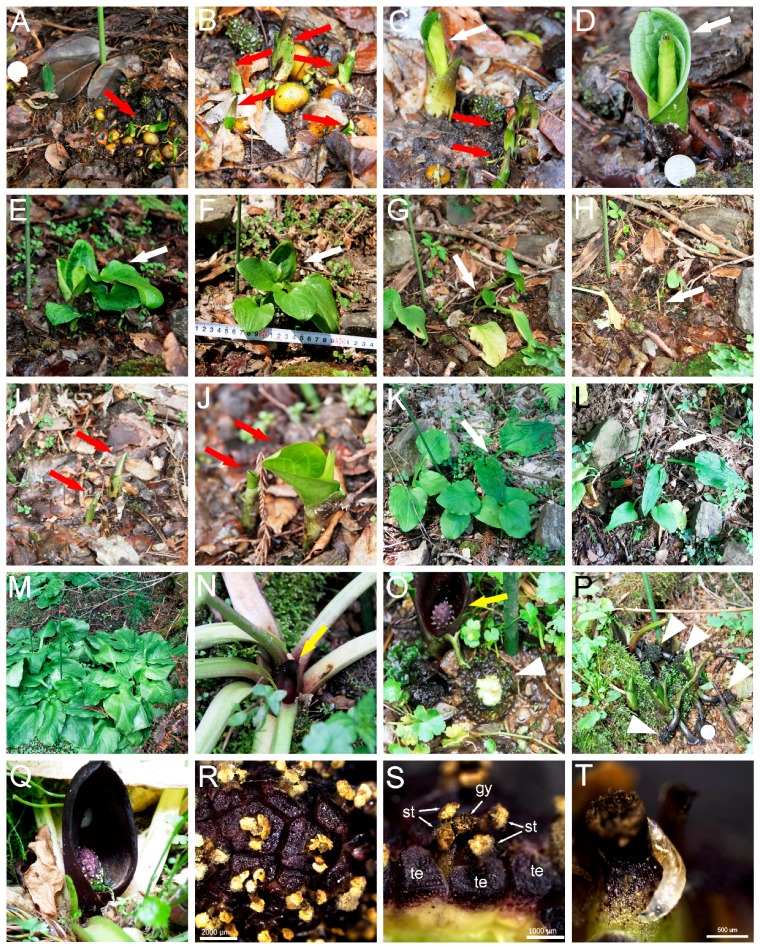
Life cycle and morphology of *S. nipponicus*. (**A**–**L**) Plants at fixed locations were traced. (**A**) Seedlings from seeds (red arrow) on 2 September 2015. (**B**) 1 January 2016 in the same region. Red arrows show seedlings from seeds. (**C**) 3 March 2016. Small seedlings from seeds (red arrows) and bigger shoot from soil (white arrow) are shown. (**D**) 15 March 2016. (**E**) 4 April 2016. (**F**) 16 April 2016. (**G**) 18 June 2016. Note that leaves turn yellow. (**H**) 4 July 2016. Leaves withered and disappeared. White arrows in (**D**) to (**H**) indicate that the shoot emerged from soil. (**I**) 13 March 2017. Two new shoots emerge from soil (red arrows). (**J**) 26 March 2017. (**K**) 18 April 2017 showing older plant expanding big leaves (white arrow). (**L**) 2 July 2017. Leaves start withering (white arrow). (**M**–**Q**) Older plants in another area in Ayabe. (**M**) 16 April 2016. Many big leaves expand. (**N**) 2 June 2016. Spathe and spadix with dark purple color emerged from the base of leaves (yellow arrow). (**O**) 18 July 2016. Spathe and spadix (yellow arrow) and fruits (white arrowhead) that had been gnawed. (**P**) Fruits diving into soil (white arrowheads). (**Q**) Spathe and spadix. (**R**) Higher magnification image of spadix with many flowers. Scale bar is 2 mm. (**S**) One flower on the spadix with four tepals, four stamens, and one gynoecium. Scale bar is 1 mm. (**T**) Maggot-like creature crawled out of flowers. Scale bar is 0.5 mm. Diameter of one JPY coin in (**A**,**D**) are 2 cm.

To examine the life cycle of *S. nipponicus*, we traced the development of the natural population in Ayabe. They grow in the understory of the forest comprising of trees such as *Cercidiphyllum japonicum* and *Cryptomeria japonica* on wet slopes, where the penetration of sunlight through the canopy is limited (Figure 1). We marked several plants in a particular area, and observed their development from 2015 to 2018. Figure 1A–L show examples of plants in one fixed area. In September 2015, they germinated from seeds, showing small leaves and roots (Figure 1A,B). In the same area, another shoot emerged from the soil, which seemed to be two or more years older than the seedlings from seeds (Figure 1C,D). This older plant expanded five green leaves in April 2016 (Figure 1E,F), and started withering in June (Figure 1G,H). Spadices or spathes did not form this year. At the same position in March next year, new shoots emerged, which are likely to be the same plants that germinated from seeds one year before (Figure 1I,J). In the same area, one that was two or more years older expanded more, had larger leaves than one year before, and did not emerge spadix or spathe in 2017 (Figure 1K,L). We found that this plant generated spadix and spathe in July 2018, suggesting that it takes more than four years to set spadices and spathes after germination from seeds.

In another area, we observed much bigger plants with many large leaves, which seemed older than the ones shown above (Figure 1M). These plants set spadix and spathe with a dark purple color at the base of the leaves (Figure 1N,O). After flowering, the spadix set small fruits, which were diving into the soil (Figure 1P). These fruits seem to mature over one year and set seeds. Near the spadix and spathe, we found a fruit that had been gnawed, probably by wild rodents (Figure 1O) [9]. In conclusion, *S. nipponicus* germinate in autumn and expand leaves the following spring; leaves wither in summer, and germinate again in the next autumn. After repetition of this life cycle for more than four years, they form spadices and spathes, and spadices develop to fruits. The fruits take another year to mature and set seeds.

The spadices of *S. nipponicus* have many small flowers, each of which consists of four tepals, four stamens, and one gynoecium (Figure 1Q–S). During the observation of spadices under stereomicroscope, maggot-like creatures crawled out of the flower interspace (Figure 1T). This suggests that the spadix of *S. nipponicus* is a habitat of small creatures, and these maggot-like creatures may be one of the pollinators of *S. nipponicus*.

### 2.2. Genetic Variation of S. nipponicus in Kyoto

**Figure 2:**
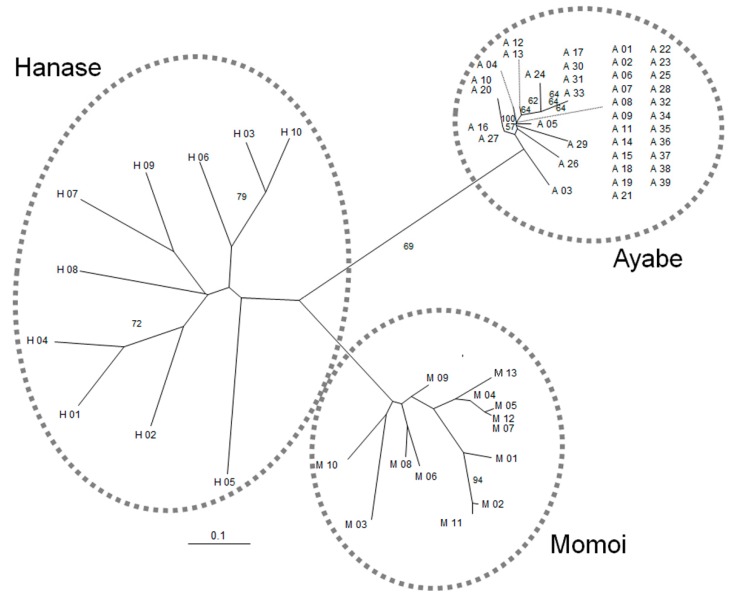
An unrooted neighbor-joining phylogram of three populations of *S. nipponicus*. Scale bar is the genetic distance based on Nei et al., 1983 [10]. Numbers at the nodes are bootstrap values from 1000 replications (>50%). Dotted circles indicate three potential groups.

To examine the genetic variation in three populations in Kyoto, we developed 11 novel microsatellite markers for *S. nipponicus* at 10 genetic loci (Table 1). The expected heterozygosity (*H_E_*) and observed heterozygosity (*H_O_*) of each marker ranged from 0.133 to 0.585, and from 0.102 to 0.243, respectively (Table 1). In the examined populations, significant deviations from the Hardy–Weinberg equilibrium were detected in only three loci of Ayabe and Momoi (Table 2). In Ayabe and Momoi, a significant or high level of the difference between *H_E_* and *H_O_* was detected at loci with *H_E_* higher than 0.1, whereas the difference was small and not significant for Hanase. Genetic distances among individuals in each population shown by a neighbor-joining (NJ) phylogram were small in Ayabe, but relatively large in Hanase (Figure 2).

**Figure 3:**
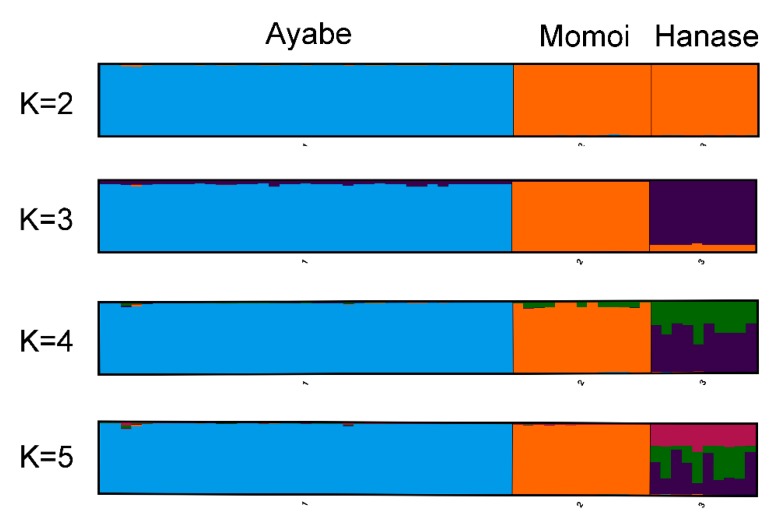
STRUCTURE analysis of *S. nipponicus* (bar plots for *K* = 2–5).

The genetic structure of these populations was examined by STRUCTURE analysis. The maximum delta *K* value was observed at *K* = 2 (Table 3), suggesting that their ancestral population was derived from two populations, and that the ancestors of Ayabe and those of Momoi and Hanase were derived from different populations (Figure 3 and Table 3).

## 3. Discussion

We unveiled the life cycle and genetic variation of *S. nipponicus*, which is one of the endangered wild plants of Araceae. The Red List categories of this species in prefectures in Japan is as follows: critically endangered (CR) and endangered (EN), Saitama, Tokyo, Yamanashi, Aichi, Kyoto, and Tottori; vulnerable (VU), Hiroshima; and near threatened (NT), Ibaragi, Tochigi, Toyama, Ishikawa, and Fukui (search system of Japanese red data: http://jpnrdb.com/index.html). Since this species grows in dim fields in the woods, we can consider several reasons for its endangerment: regional development for residence, the afforestation of *C. japonica* that could have changed the ecosystem around the habitat, or recent climate change such as global warming. Also, significant deviation from the Hardy–Weinberg equilibrium in Ayabe and Momoi suggests that there are some modes of non-random mating such as inbreeding and population subdivision within these populations.

We found that *S. nipponicus* takes more than four years to set flowers, since they germinate from seeds. During vegetative growth for a couple of years, they may accumulate photosynthesized carbohydrates in roots, and afterwards, they enter the reproductive phase to set seeds. The plant germinates in autumn, expands leaves in spring, and generates inflorescence consisting of dark purple spathe and spadix (Figure 1). This seasonal life cycle and the timing of flowering differ among the *Symplocarpus* species growing in Japan: *S. renifolius* generates inflorescence in winter with melting snow around them, and afterwards expands leaves, while in *S. nabekuraensis*, leaves and inflorescence emerge simultaneously in the summer [1]. This suggests that each species has adapted to the ambient environment. *S. renifolius* generates heat in spadices in a stage-dependent manner, which is proposed to spread odor to attract pollinators, promote flowering, protect from freezing, and/or assist pollen germination and pollen tube development [11,12], but we do not know whether *S. nipponicus* and *S. nabekuraensis* carry the heat-generation ability in their spadices, since the summer is their flowering season.

For conservation of the endangered species, one of the first things to do is to grasp the genetic diversity of a population. Our results offered the endangered level of each population and genetic diversity among populations. The SSR markers that we developed can be used for analyzing the genetic diversity of other populations, since they are PCR-based markers, and are thus relatively easy to use. The low genetic diversity in the Ayabe population would be a result of the habitat fragmentation, since the isolation of a habitat causes a loss of genetic variability; particularly rare species carries less genetic diversity than common species [13,14]. Populations in Momoi and Hanase showed relatively higher genetic variation, indicating that their natural environment is more conserved. The use of microsatellite markers allows us to understand the current status of the population and guess the history of what happened to habitats before. Individuals from the Momoi and Hanase populations, which are closely located to each other, were completely diverged in our phylogenetic analysis. This result indicates that these populations are genetically isolated, and migration rarely occurs between them. There are several ways to conserve the local populations. One is to maintain the local population by transplanting, but neither our trial for seed germination nor transplanting with horticultural soil has worked so far, which is probably due to the special requirements of the living environment of *S. nipponicus*. Another is to transplant plants living in the other population, but this will cause the crossing of different genotypes. Also, some residents of the local villages in Japan do not wish to bring in ecotypes from other regions. To maintain the genetic resources of local areas, we should keep our eyes on the status of the plants growing in these populations over years, including checking the genetic diversity by microsatellite markers at some points.

In conclusion, we revealed the life cycle and genetic diversity of *S. nipponicus* in Kyoto. The microsatellite markers can be used to assess the genetic variation in the other populations. We hope that our results help conserve this endangered species, and that more people pay attention to the many organisms that are disappearing due to our activities.

## 4. Materials and Methods

### 4.1. Populations of S. nipponicus

**Figure 4:**
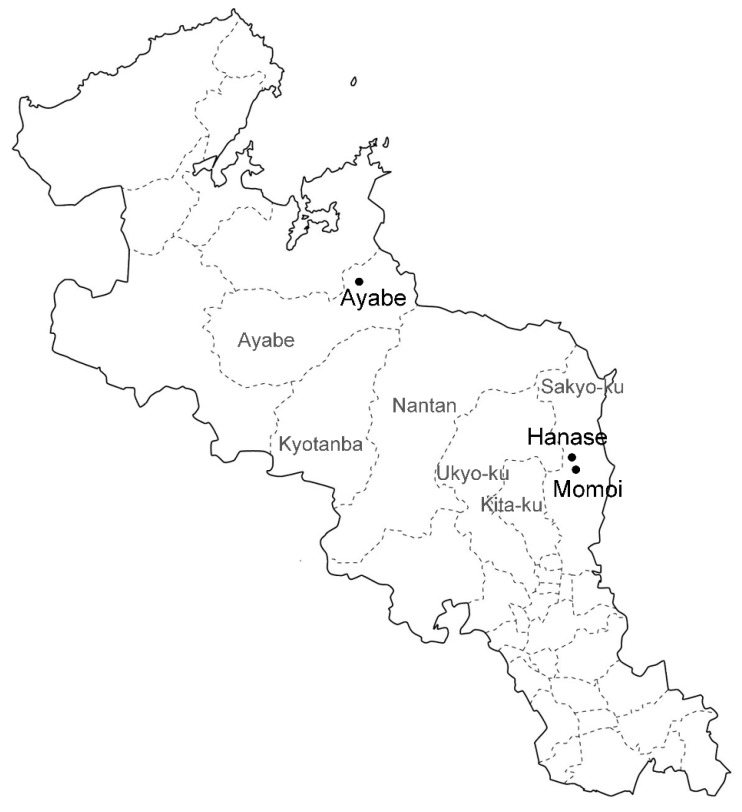
Location of the three populations of *S. nipponicus* used in this study in Kyoto Prefecture. Population areas are shown by black dots and letters. Gray letters indicate the municipality around the populations.

Autogenous *S. nipponicus* plants growing in Ayabe (35°24′ N, 135°27′ E, approximately 200 m above sea level), Hanase (35°21′ N, 135°77′ E), and Momoi (35°22′ N, 135°82′ E) in Kyoto Prefecture were used (Figure 4). Plants in Ayabe were pictured every month to trace the development from August 2015 to June 2018. Photos were taken with a Canon EOS 7D (Canon, Tokyo, Japan) and S8AP0 stereomicroscopy mounted with an EC3 digital camera (Leica, Wetzlar Germany).

### 4.2. DNA Extraction and Development of Microsatellite Markers

Leaf tissues of 62 individuals were collected from three habitats (Ayabe, *n* = 39: Momoi, *n* = 13: and Hanase, *n* = 10). Total DNA was extracted from a leaf by DNeasy Plant Mini kit (QIAGEN, Hilden, Germany). Microsatellite-enriched libraries were constructed from the total DNA of an individual from Ayabe using (CA)_15_ or (GA)_15_ biotin-labeled oligonucleotide (Invitrogen, Carlsbad, CA, USA) [15]. A total of 64 clones containing 200–900 bp inserts were selected and sequenced. Twenty-two clones containing microsatellites were selected for primer design flanking the SSR repeats. All of the primers were tested for PCR amplification using two individuals of *S. nipponicus*.

### 4.3. Phylogenetic Analysis

PCR was performed with KAPA Taq EXtra PCR Kit (Nippon Genetics, Tokyo, Japan) under the following conditions: initial denaturation at 94 °C for 1 min, 30 cycles of 94 °C for 30 s, 50 °C for 30 s, and 72 °C for 1 min, and the final extension at 72 °C for 5 min. Twelve primer sets that were successful in the PCR were selected for the genotyping of *S. nipponicus* populations. The fragment analysis of the PCR products was performed for the 62 individuals with fluorescent-labeled forward primer (Alpha ADN, Montreal, QC, Canada) and non-fluorescent reverse primer. The PCR products were analyzed with a CEQ8000XL DNA sequencer (AB Sciex, Tokyo, Japan) for scoring the fragment size of each locus. Finally, 11 primer pairs were selected as polymorphic microsatellite markers. The expected and observed heterozygosity (*H_E_* and *H_O_*), fixation index (*F_IS_*) in each population, and Hardy–Weinberg equilibrium test were calculated by Genepop version 4.2 [16]. The number of alleles (*N_A_*) in each population, and *N_A_*, *H_E_*, *H_O_* and *F_IS_* for each locus were analyzed by GenAlEx 6.5 [17]. Phylogenetic relationships among individuals were inferred by the neighbor-joining (NJ) method [18] using Populations 1.2.32 (http://www.bioinformatics.org/project/?group_id=84) based on Nei’s *D_A_* genetic distance [10]. Population structure was analyzed by the Bayesian clustering method using STRUCTURE 2.3.4 [19,20]. Markov chain Monte Carlo simulations were run using the admixture model assuming correlated allele frequencies among the populations. Ten independent runs were performed with each *K* value (ranging from 1 to 5). The length of each run was 1,000,000 iterations following 100,000 burn-ins. Delta *K* [21] was adopted as the criterion for the choice of appropriate *K* value.

## Figures and Tables

**Table 1:** Characteristics of novel microsatellite markers in *Symplocarpus nipponicus*.

Locus	Primer Sequence (5′→3′)	*T_m_* (°C)	Repeat Motif	Allelic Size Range (bp)	*N_A_*	*H_E_*	*H_O_*	*F_IS_*	Accession Number
SniAlu_CA08-1	F:TAAGGGAGTTGATATCACTACC R:CACCTTCATGATAATAGTATAGGGT	55	(CT)_15_	145–149	2.000	0.180	0.126	0.301	LC342874 *
SniAlu_CA08-2	F:TAAGGGAGTTGATATCACTACC R:CACCTTCATGATAATAGTATAGGGT	55	(CT)_15_	153–193	2.333	0.198	0.167	0.160	
SniAlu_CA12	F:ATCCTGACATTGACATCCCAC R:ATACATTGCGTACAGAACACAAATAC	56	(CA)_16_	173–187	2.667	0.294	0.192	0.345	LC342875
SniAlu_CA14	F:ATGTGTGCATGTGCCCTTTAAC R:GTGTTCTTCTATGCACATGTAGG	57	(TG)_14_	202–217	2.667	0.268	0.192	0.282	LC342876
SniAlu_CA15	F:GACTTTGCTTTGTGAACACATGG R:TGGTGATACTTTATGTGGTGCCT	57	(AG)_24_	142–171	2.667	0.257	0.184	0.284	LC342877
SniAlu_CA28	F:GGCCTTCTCCACGCTAAAAT R:TAAGCCATAATTGCCGACAC	56	(CT)_16_	142–155	1.667	0.133	0.109	0.187	LC342878
SniAlu_CA36	F:CTTCCAAATAACAAAGAGTGTCCAC R:CTTCCATCGCCATCATCATATC	57	(GA)_23_	163–189	2.667	0.376	0.177	0.530	LC342879
SniAlu_GA02	F:GGGTAGGACAAGGCATCAATA R:CAATGGGGGATCTTTTTTAGGG	57	(AG)_25_(CCCCATCAC)1(AG)_28_	175–231	5.333	0.585	0.243	0.585	LC342880
SniAlu_GA04	F:TTCTGGAGGCTTTCTCTTCATG R:GCAATTCCTGTCAAGGTATCAATG	57	(CT)_9_(GTCTGT)_1_(CT)_2_(T)_1_(CT)_7_	207–213	1.667	0.207	0.200	0.032	LC342881
SniAlu_GA08	F:GGAAGGGTATAGGCAATTTGTAGG R:TGATCGTTAAGGTCGTCAACCA	57	(GA)_9_,AA(GA)_10_	144–152	2.333	0.195	0.102	0.478	LC342882
SniAlu_GA15	F:CATGATATCACTCTCCCTGTTC R:CACTAGTTACATGCTTCCACTTG	57	(CT)_27_	123–153	2.667	0.322	0.218	0.323	LC342883
Mean					2.606	0.274	0.174	0.319	

*T_m_*, optimized annealing temperature; *N_A_*, average number of alleles across population; *N_E_*, number of effective alleles; *H_E_*, expected heterozygosity; *H_O_*, observed heterozygosity; *F_IS_*, fixation index; * Derived from the same primer set producing two different amplicons.

**Table 2:** Genetic characteristics of 10 polymorphic microsatellite loci for three populations of *S. nipponicus* in Kyoto.

Locus	Ayabe (*n* = 39)				Momoi (*n* = 13)				Hanase (*n* = 10)			
	*N_A_*	*H_E_*	*H_O_*	*F_IS_*	*N_A_*	*H_E_*	*H_O_*	*F_IS_*	*N_A_*	*H_E_*	*H_O_*	*F_IS_*
SniAlu_CA08-1	1.000	0.000	0.000	N.A. ^a^	2.000	0.077	0.077	0.000	3.000	0.500	0.300	0.400
SniAlu_CA08-2	1.000	0.000	0.000	N.A. ^a^	1.000	0.000	0.000	N.A. ^a^	5.000	0.633	0.500	0.211
SniAlu_CA12	1.000	0.000	0.000	N.A. ^a^	2.000	0.333	0.077	0.769	5.000	0.606	0.500	0.174
SniAlu_CA14	1.000	0.000	0.000	N.A. ^a^	2.000	0.077	0.077	0.000	5.000	0.783	0.500	0.362
SniAlu_CA15	2.000	0.051	0.051	−0.013	1.000	0.000	0.000	N.A. ^a^	5.000	0.772	0.500	0.353
SniAlu_CA28	2.000	0.026	0.026	0.000	1.000	0.000	0.000	N.A. ^a^	2.000	0.400	0.300	0.250
SniAlu_CA36	1.000	0.000	0.000	N.A. ^a^	4.000	0.785	0.231	0.706 ***	3.000	0.422	0.300	0.290
SniAlu_GA02	4.000	0.279	0.051	0.816 ***	5.000	0.731	0.077	0.895 ***	7.000	0.861	0.600	0.303
SniAlu_GA04	1.000	0.000	0.000	N.A. ^a^	1.000	0.000	0.000	N.A. ^a^	3.000	0.656	0.600	0.085
SniAlu_GA08	3.000	0.192	0.051	0.733 ***	2.000	0.147	0.154	−0.044	2.000	0.278	0.100	0.640
SniAlu_GA15	1.000	0.000	0.000	N.A. ^a^	2.000	0.455	0.154	0.662	5.000	0.572	0.500	0.126

^a^, Not applicable; Asterisks, significant deviations from the Hardy–Weinberg equilibrium after Bonferroni’s correction (***; *p* < 0.001).

**Table 3:** STRUCTURE analysis.

*K*	Reps	Mean LnP (*K*)	Stdev LnP (*K*)	Delta *K*
1	10	−1393.9200	0.5073	N.A. ^a^
2	10	−731.0800	1.5194	303.032579
3	11	−528.6545	64.19127	3.247736
4	10	−534.7100	10.7532	1.580497
5	10	−523.7700	12.5947	NA

^a^: Not applicable.
